# High-Protein Nutritious Flatbreads and an Option for Gluten-Sensitive Individuals

**DOI:** 10.3390/foods8110591

**Published:** 2019-11-19

**Authors:** Talwinder S. Kahlon, Roberto J. Avena-Bustillos, Jenny L. Brichta, Ashwinder K. Kahlon

**Affiliations:** Western Regional Research Center, USDA-ARS, 800 Buchanan St., Albany, CA 94710, USATalwinder2425@gmail.com (A.K.K.)

**Keywords:** Quinoa, wheat, peanut oilcake, broccoli, beets, whole grain, flatbreads

## Abstract

Whole grain quinoa and wheat, high-protein vegetable flatbreads were evaluated by tasters and a physical analysis was conducted. The objective was to produce nutritious, tasty gluten-free (quinoa) as well as gluten-containing (wheat) flatbreads. Flatbreads were Quinoa Peanut Oilcake Broccoli (QPCBROC), Wheat Peanut Oilcake Broccoli (WPCBROC), Quinoa Peanut Oilcake Beets (QPCBEET) and Wheat Peanut Oilcake Beets (WPCBEET). Peanut Oilcake would increase protein and add value to this farm byproduct. Bile acid binding broccoli and beets with cholesterol-lowering potential were used. Tasters preferred QPCBROC flatbreads for all sensory parameters. Acceptance of flatbreads was QPCBROC (83%), WPCBROC (70%), QPCBEET (78%) and WPCBEET (69%); these values were statistically similar. The objective of ≥25% protein content was exceeded by 5–8% and that of ≥70% acceptance was adequately achieved. These flatbreads were low in fat (5–6%) and contained essential minerals (4%) with only ≤1% added salt. Porosity and expansion data suggest that these flatbreads would take up relatively little shelf space. These flatbreads require only three ingredients and can be made in a household kitchen or by commercial production. These flatbreads offer a nutritious, tasty choice for all, and quinoa flatbreads offer an option for gluten-sensitive individuals.

## 1. Introduction

Flatbreads are the oldest of all bread products and are consumed worldwide. Wheat is a common ingredient in most flatbreads [1]. Flatbreads are commonly baked on a griddle (roti or chapati) or in an oven (naan) or pan fried (paratha) or deep-fat fried (puri or bhatura). They can be served with soups, curries, vegetables and meat dishes, and can be used as a dish or spoon to scoop up other portions of the meal. Cultures regularly consuming flatbreads, on an average, would consume one paratha for breakfast, two chapatis for lunch and three chapatis for dinner. Hard red winter wheat flour chapatis and parathas of medium size were cooked using 45 g and 75 g flour, respectively, thus daily average flour consumption would be (45 × 5 + 75) = 300 g. Various fast food chains, food markets and airlines have introduced flatbread wraps and sandwiches. The American Baking Society has conducted a Flatbread Product Development contest, awarding several scholarships [2]. The U.S. Department of Agriculture (USDA) Dietary Guidelines for Americans 2015–2020 recommend consuming at least one-half of dietary intake of whole grains such as quinoa (gluten-free) and wheat (gluten-containing) [3]. Most children and elderly do not meet their protein and vegetable intake requirements [4].

Flatbread protein content can be increased with food grade, low-value, farm by-products, like peanut oilcake. Use of vegetables, such as broccoli and beets, that contain healthful phytonutrients, should be encouraged. The cholesterol-lowering potential of various vegetables (turnips, cabbage, cauliflower, bell pepper, spinach, asparagus, green beans, mustard greens, broccoli, egg-plant, carrots, brussels sprouts, collard greens, kale, okra and beets), as determined by their bile acid binding relative to cholestyramine (cholesterol lowering bile acid binding drug), has been reported [5,6,7,8,9,10].

Consumption of whole grain products has been shown to lower the risk of many preventable lifestyle degenerative diseases [11,12]. About 1–3% of the world population has celiac disease or some degree of gluten-sensitivity. These individuals must follow a gluten-free diet, which restricts their wheat-containing options [13]. Celiac disease results in erosion of the lining of small intestines [14,15].

In previous studies, gluten-free whole grain corn, sorghum, brown rice and millet flatbreads had acceptance rates of 83%, 79%, 77% and 50% respectively [16]. Acceptances for whole grain quinoa, teff, amaranth and buckwheat (with hulls) were reported as 84%, 72%, 66% and 38% respectively [17]. Also, buckwheat (without hulls) peanut oilcake and beet flatbreads (BPB), and those with additions of onion, garlic or ginger, had acceptance rates of 66–79% [18]. Quinoa peanut oilcake kale (QPK), and those with added onion, garlic or cilantro, have been reported having 72–92% acceptance rates [19].

Acrylamide is formed during thermal processing of plant-based foods such as cereals and potatoes, foods that are widely consumed. Acrylamide has been reported to cause adverse effects in animals and possibly in humans, including carcinogenicity, neurotoxicity, teratogenicity, and anti-fertility. When cooked at lower temperature for a longer time, significantly lower acrylamide production has been observed in quinoa flatbreads (6–7 mg/kg) and wheat flatbreads (21–29 mg/kg) [20,21]. In the study reported herein, that cooking method was adopted.

The objective of this study was to compare sensory evaluation of gluten-free quinoa with gluten containing wheat in order to provide gluten-free flatbreads for gluten sensitive individuals and whole wheat flatbreads for rest of the population. Consumers need to be educated about the benefits of preparing nutritious tasty flatbreads at home and finding those available in food markets.

The aim was to achieve ≥70% acceptability and ≥25% protein of healthy, nutritious, tasty flatbreads. The flatbreads were Quinoa Peanut Oilcake Broccoli (QPCBROC), Wheat Peanut Oilcake Broccoli (WPCBROC), Quinoa Peanut Oilcake Beets (QPCBEET) and Wheat Peanut Oilcake Beets (WPCBEET). Flatbreads containing quinoa or wheat, with peanut oilcake and broccoli or beets, have not been reported previously, nor are they available commercially.

### 1.1. Quinoa

Quinoa (*Chenopodium quinoa*) is a round, disc-shaped pseudocereal. Quinoa is gluten-free and cooks like rice. The top three quinoa-producing countries (Peru, Bolivia and Ecuador) produced 192.5 metric tons in 2014 [22]. After harvesting, the quinoa grain is processed to remove the saponin-containing outer coating. It is considered an ideal food, as it contains all nine dietary essential amino acids (histidine, isoleucine, leucine, lysine, methionine, phenylalanine, threonine, tryptophan and valine). Quinoa is a good source of dietary fiber, minerals and unsaturated fatty acids. The Food and Agricultural Organization of the United Nations (FAO) declared 2013 “The International Year of the Quinoa” [23]. FAO intended to focus world attention on the role that quinoa can play in providing food security and nutrition, and in the eradication of poverty. Quinoa, at about $5 per kg, is less affordable than whole wheat flour, currently at less than $2 per kg.

### 1.2. Wheat

World production of wheat (*Triticum aestivum*) is estimated to be 765 million metric tons in 2019–2020 [24]. Top wheat-producing countries in million tons per year are the European Union 152, China 132, India 102, Russian Federation 73 and USA 53. Wheat contains gluten that has viscoelastic and adhesive properties which facilitate production of bread with uniform honeycomb structure, as well as pasta, snacks and many other foods. When eaten as whole grain, wheat is a source of multiple nutrients and dietary fiber.

### 1.3. Peanuts

Peanuts (*Arachis hypogaea*), belonging to the botanical family Fabaceae, are also known as Leguminosae. In the U.S., peanuts are mostly used in food and confectionary products, but more than 50 percent of the world’s production is crushed for oil. Top peanut-producing countries in million tons per year are China 16.7, India 6.9, Nigeria 3.0, USA 2.6 and Sudan 1.8. World production of peanuts is over 45 million metric tons per year [25]. Nearly 24 million tons of peanuts are used to extract oil, resulting in 12 million tons of peanut oilcake. It is a low value byproduct mainly used as animal feed. On a dry matter basis, peanut oilcake contains 50% protein (derived from Table 1).

### 1.4. Broccoli

Broccoli (*Brassica oleracea*) belongs to the cabbage family. It is a nutritious common green vegetable. Global production of broccoli was 27 million tons in 2017 [26]. China is the top broccoli-producing country, followed by India and US. California produces 90% of domestic broccoli. Broccoli is eaten raw or cooked by boiling, steaming, microwaving or stir-frying. Steamed broccoli binds significantly more bile acid than uncooked (5, 9). It is rich in vitamins C and K. It contains health-promoting sulfur compounds, glucosinolates, isothiocyanates and sulforaphane.

### 1.5. Beets

Beets (*Beta vulgaris*) have been grown for food since ancient times. Both the leaves and the root are edible [27]. Beets are also a rich source of alkaloid betaine, as well as B-vitamin folate. The betaine and folate together lower blood homocysteine. In addition, it is a moderate source of iron, potassium, vitamin C and fiber. Beets are rich in nitrates, which the body converts to nitric oxide, a compound that relaxes and dilates blood vessels, resulting in better circulation and possibly lowering blood pressure. It has been reported that beets bind significantly more bile acids than many other vegetables [6,8].

## 2. Materials and Methods

### 2.1. Materials and Composition

Kirkland Signature quinoa was obtained from Costco. Quinoa flour was prepared using Blendtec Kitchen Mill Model 91 at medium setting (Blendtec Inc., Wichita, KS, USA). Whole wheat flour was obtained from Bob’s Red Mill (Milwaukie, OR, USA). Peanuts, broccoli and beets were purchased from local food markets. Peanut oilcake was produced by extracting oil using Vevor Oil Press (Joyfay.com, Cleveland, OH, USA). Peanut oilcake was ground and broccoli and beets were chopped using Mini-Prep Processor (Cuisinart.com, East Windsor, NJ, USA). Composition of quinoa flour, wheat flour, peanut oilcake, broccoli and beets is given in Table 1. Dough composition of the flatbreads, tested on an as-is basis, was quinoa flour, wheat flour and peanut oilcake (36%), broccoli and beets (27%), salt (0.74%), and is given in Table 2.

### 2.2. Flatbread Making Process

Flatbread dough was prepared by adding 36–54 mL water to 100 g of as-is ingredients. Formulation of each of the four kinds of flatbreads for best taste was decided by the consensus of laboratory personnel. Water was added slowly in small volumes to flatbread ingredients until the dough began forming a ball. The dough was kneaded until it became smooth and elastic, which determined the proper amount of water needed for each batch of flatbreads. Dough was placed in a Pyrex bowl, covered with a polyvinyl film and held at room temperature for 30 min. 50 g of dough was put on parchment paper (nonstick, oven-safe up to 216 °C) and pressed into a thickness of 1–1.5 mm and to a roughly 17 cm diameter circle in a 20 cm Alpine Cuisine flatbread Press (Aramco Imports, Inc., Commerce, CA, USA). Flatbreads were cooked between upper and lower hot irons of the flatbread maker for 2 min (1 min each side) at 165–195 °C on parchment paper in a 1000 Watts CucinaPro Flatbread Maker (SCS Direct, Inc., Trumbull, CT, USA). For sensory evaluation, uncooked flatbreads were frozen under nitrogen in Ziploc bags. On the day of testing, flatbreads were thawed and cooked. For proximate and physical analysis, flatbreads were cooked immediately. Cooked flatbreads were chopped for 30 sec in a Cuisinart coffee grinder Model DCG-20N (Cuisinart East Windsor, NJ, USA). Chopped broccoli, beets and flatbreads were dried at 103 °C for 3 h and confirmed with additional 1 h drying. Dried broccoli, beets and flatbreads were ground to fine powder using a coffee grinder (Cuisinart Model DCG-20N E Windsor NJ, USA). Ingredients (quinoa flour, wheat flour, peanut oilcake, broccoli, beets) and flatbreads were analyzed for nitrogen using AOAC method 990.03 [28] by Leco FP628 analyzer (Leco Corporation, St Joseph, MI, USA); for crude fat by Soxhlet extraction with petroleum ether using AOAC method 27.006 [29]; for ash using AOAC method 923.03; and for moisture using AOAC method 935.29 [30].

The cooking temperature ranged from 165–195 °C, as measured by Fluke 61 Infrared Thermometer (www.fluke.com). For crispier or chewier flatbreads, cooking time can be adjusted up or down as desired, according to personal preference. The pliable flatbreads could be filled and rolled to make wraps.

### 2.3. Sensory Evaluation

**Figure 1:**
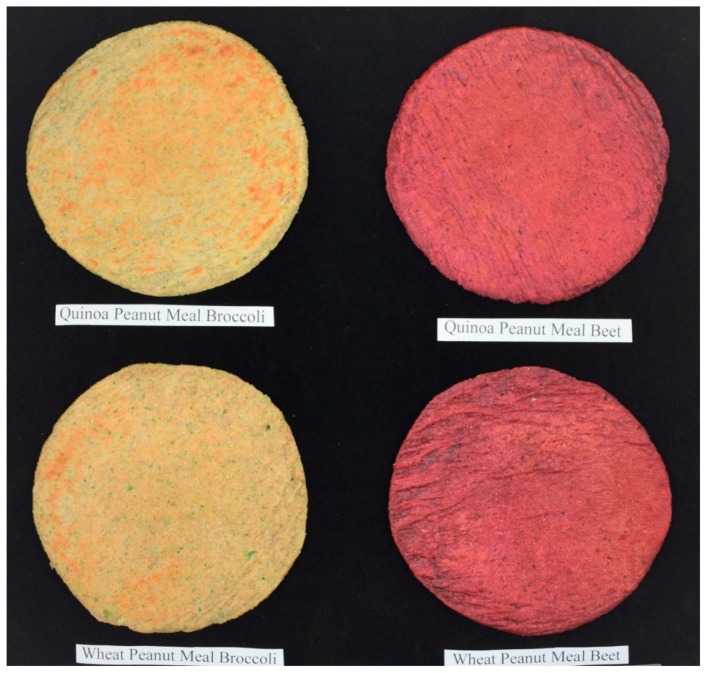
Cooked Flatbreads.

**Figure 2:**
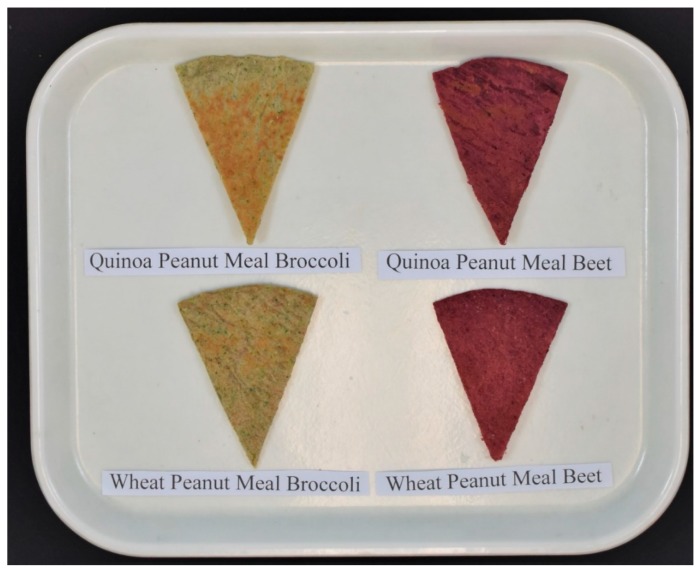
Flatbread samples as presented for sensory evaluation, *n* = 80.

Cooked whole grain, high-protein, vegetable flatbreads are shown in Figure 1. Freshly cooked flatbreads were kept in insulated containers before presenting for sensory evaluation. Flatbreads were cut into eight sectors with a pizza cutter and all four kinds of flatbread samples were presented appropriately labelled to tasters, as shown in Figure 2. Evaluation was conducted in individual sensory booths under white light. The tasters (*n* = 80) were instructed to evaluate sensory parameters for each flatbread sample individually and not on a relative scale. The evaluation of flatbreads for Color/Appearance, Odor/Aroma, Taste/Flavor, Texture/Mouth-Feel was performed on a scale of 1–5 (dislike very much = 1, dislike slightly = 2, neutral = 3, like slightly = 4, like very much = 5). The overall acceptable preference was on a scale of 1–2 (not acceptable = 1, acceptable = 2).

### 2.4. Physical Analysis

#### 2.4.1. Water Activity

Water activity of flatbreads was measured at 25.01 ± 0.02 °C in triplicate using an AquaLab 4TE dew point water activity meter (Decagon Devices, Inc., Pullman, WA, USA).

#### 2.4.2. True Density

True density (ρt) of the whole grain, high-protein, vegetable flatbreads was determined using gas displacement pycnometer AccuPyc II 1340 (Micromeritics Instrument Co., Norcross, GA, USA) at 21.4 ± 0.4 °C. Samples of flatbreads were dried in triplicate at room temperature for 15 h at 0% relative humidity in a vacuum desiccator with anhydrous calcium sulfate (W.A.Hammond Drierite, Xenia, OH, USA). After drying, small pieces of sample were compressed into a density-measuring cylinder of the pycnometer and fifteen true density measurements were recorded.

#### 2.4.3. Bulk Density

The bulk density (ρb) of quinoa, wheat, peanut oilcake, broccoli and beet flatbreads was measured using Syntron Vibra-Flow, Model F-T01 (Syntron Company, Homer City, PA, USA). In a 202 mL volume jar, displacement by 10 g flatbread sample in Ottawa Sand was measured. The first reading was taken after shaking for 15 min; a subsequent two readings were taken after additional shaking for 5 min each time (*n* = 15).

#### 2.4.4. Texture Analysis

The texture of the flatbreads was determined with a TA.XT2 Plus (Texture Technologies Corp., Hamilton, MA, USA) using a TA-108N Large Film Extensibility Rig. The Large Film Extensibility Rig is an acrylic cylinder with a 63 mm diameter opening mounted on an aluminum platform. Each flatbread was pre-punched with four holes to fit over the uniform alignment pins. The test samples were held tightly with alignment pins between plated with similar openings. The platform held the flatbreads with uniform tension. The probe was a TA-108N 7/16″ diameter acrylic cylinder, which has a radius edge to minimize any cutting of the moist flatbreads. The probe traveled at 1.7 mm/s until the flatbread surface was detected at 20 g force. The probe then traveled down at the same speed for up to 10 mm, a distance chosen to stretch all the flatbreads until they thoroughly ruptured. The probe withdrew at the same speed, as well. The test was designed to measure flatbreads’ stretchability, breaking point and firmness in gm. All products were tested at room temperature. Each test was conducted 10 times.

#### 2.4.5. Color Analysis

A Minolta CM-3500d (Konica Minolta Sensing Americas, Inc., Ramsey, NJ, U.S.A.) spectrophotometer was used to find the samples’ color on the *L*∗ (D65), *a*∗ (D65), and *b*∗ (D65) scales. The spectrophotometer was fitted with a CM-A196 Target Mask 8 mm diameter (Konica Minolta Sensing Americas, Inc., Ramsey, NJ, USA) and the sample was measured in five zones directly over the target opening.

### 2.5. Statistical Analysis

Statistical analysis of quinoa, wheat, peanut oilcake, broccoli and beet flatbreads were evaluated with sensory parameters on a scale of (1–5) and acceptability data on a scale (1–2) for all tasters. Results were analyzed with Minitab software (version 14.12.0, Minitab Inc., State College, PA, USA), using basic statistics for mean ± SEM and one-way analysis of variance, and Tuckey’s multiple comparison tests with (*p* ≤ 0.05) was considered the criterion of significance. In order to determine statistical differences among flatbread samples, significant standardized range at 5% level, using multiple range test, was conducted.

## 3. Results and Discussion

### 3.1. Sensory Evaluation Analysis

Eighty in-house volunteers (45 women and 35 men) in individual sensory booths under white light, using approved methods, judged Color/Appearance of flatbreads quinoa peanut oilcake broccoli (QPCBROC), quinoa peanut oilcake beets (QPCBEET) and wheat peanut oilcake beets (WPCBEET) to be significantly (*p* ≤ 0.05) better than wheat peanut oilcake broccoli (WPCBROC) (Table 3). The Odor/Aroma of QPCBROC was significantly higher than WPCBROC and WPCBEET flatbreads. Values were also significantly higher for QPCBEET than WPCBEET. Taste/Flavor of the QPCBROC was significantly preferred to that of QPCBEET and WPCBEET. Texture/Mouth feel of the QPCBROC was significantly higher than WPCBEET. Acceptance of quinoa and wheat flatbreads with broccoli or beets was judged to be similar by the tasters. QPCBROC flatbreads were preferred for all sensory parameters.

### 3.2. Acceptability

**Figure 3:**
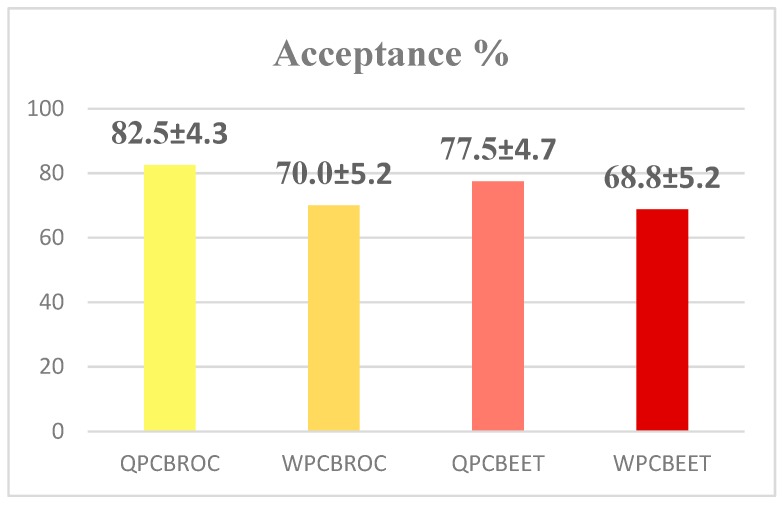
Acceptance of Flatbreads Quinoa Peanut Oilcake Broccoli (QPCBROC), Wheat Peanut Oilcake Broccoli (WPCBROC), Quinoa Peanut Oilcake Beets (QPCBEET) and Wheat Peanut Oilcake Beets (WPCBEET), *n* = 80. There were no significant differences in acceptance of flatbreads tested (*n* = 80).

Percent acceptance of the quinoa wheat peanut oilcake broccoli and beet flatbreads is given in Figure 3. Acceptance of flatbreads tested was QPCBROC (83%), WPCBROC (70%), QPCBEET (78%) and WPCBEET (69%). Acceptance values for the four kinds of flatbreads tested were statistically similar. QPCBROC and QPCBEET acceptance exceeded goal by 13% and 8%, respectively. The objective of attaining acceptability ≥70% was adequately achieved.

### 3.3. Physical Analysis

Water activity (Aw) of the QPCBROC, WMPBROC and WPCBEET was similar, and significantly higher than QPCBEET (Table 4). Values ranged from 0.92–0.94. Most spoilage molds, yeasts and bacteria can grow on foods with an Aw of 0.80, 0.88 and 0.90, respectively [31]. Food products with higher Aw’s than flatbreads tested, such as cheese (0.97), salad dressing (0.97–0.98), and fresh meat (0.98), are perishable and need refrigeration [32]. The Aw of flatbreads tested suggest they were chewy and should preferably be served freshly cooked. They could be stored in a refrigerator for up to three days. Similar uncooked flatbreads were vacuum sealed after flushing with nitrogen and stored in the freezer using Ziploc bags. After six months, these flatbreads were thawed to room temperature and cooked as described here. No adverse effects in sensory parameters were observed by consensus of the laboratory personnel. True density (ρt) of the flatbreads tested were similar and the values ranged from 1.40 to 1.44. Bulk density (ρb) of QPCBROC was significantly lower and porosity significantly higher than WPCBROC, QPCBEET and WPCBEET flatbreads. A porosity of (0.33–0.44) and expansion of (1.50–1.81) suggest that these flatbreads would take up a relatively small shelf space.

Break force for beet-containing flatbreads were significantly higher than those with broccoli. Break force for QPCBEET was also significantly higher than WPBBEET. Data suggest that beet-containing flatbreads were tougher and with lower porosity, potentially due to higher fiber in beets than in broccoli. Stretchability for QPBBROC, WPCBROC and WPCBEET was similar and significantly higher than QPCBEET. Wheat-containing flatbreads are expected to be higher in stretchability, due to the elasticity of gluten. However, highest stretchability and porosity were found in QPCBROC, along with lowest break force, which was unexpected and is difficult to explain.

Color values, as L* (black = 0 and white =100), a* (green −, red +) and b* (blue −, yellow +), were significantly different from each other in the flatbreads tested (Table 5). Wheat-containing flatbreads WPCBROC as well WPCBEET imparted L* significantly lighter in color than corresponding QPCBROC and QPCBEET, respectively. Relative a* values for WPCBEET were significantly higher towards red than QPCBEET, whereas QPCBROC values were higher than WPCBROC. Relative b* for QPCBROC and QPCBEET were significantly higher towards yellow than wheat-containing flatbreads (WPCBROC and WPCBEET).

The composition of cooked whole grain, high-protein, vegetable flatbreads is given in (Table 6). These flatbreads, on a dry matter (DM) basis, contained protein 30–33%, crude fat (5–6%), ash (4.13–4.40%), dietary fiber (9–11%), carbohydrate (57–61%), DM (65–69%) and water (31–35%). The protein, as well as mineral content, of the flatbreads tested were significantly different from each other; the narrow range of these values indicates high precision in these analytical methods. The objective to attain a final product with 25% protein on DM basis was exceeded by 5–8%. The mineral content of the flatbreads tested was 4%, with less than 1% added salt. These flatbreads were low in unhealthy sodium, salt and fat.

### 3.4. Conclusions

Sensory evaluation and physical testing were conducted on Quinoa Peanut Oilcake Broccoli (QPCBROC), Wheat Peanut Oilcake Broccoli (WPCBROC), Quinoa Peanut Oilcake Beets (QPCBEET) and Wheat Peanut Oilcake Beets (WPCBEET) flatbreads. The objective was to have nutritious gluten-free, as well as gluten-containing, flatbreads, with ≥25% protein and ≥70% acceptability. Protein content of evaluated flatbreads was 25–33%; this goal was clearly met. Acceptance of flatbreads tested ranged from 69–83%. The Quinoa Peanut Oilcake Broccoli flatbread was preferred in all sensory parameters over the other three by 80 inhouse evaluators. As quinoa offers all the dietary essential amino acids, it would be considered more nutritious. Since the acceptability was statistically similar among the flatbreads tested, all the products were equally desirable. Porosity and expansion data suggest that all tested flatbreads would take up relatively little shelf space. These flatbreads require only three ingredients and can be made in any household kitchen or by commercial production. These flatbreads were low in fat and sodium. They offer a nutritious, tasty choice to all, and quinoa flatbreads are suitable for gluten sensitive individuals.

## 4. Statement

USDA is an equal opportunity provider and employer. Mention of trade names or commercial products in this report is solely for the purpose of providing specific information and does not imply recommendation or endorsement by the U.S. Department of Agriculture.

## Figures and Tables

**Table 1:** Chemical Composition of Raw Materials (%).

Ingredients	Protein	Fat	Minerals	Dietary Fiber	Carbohy-Drates	DM	Water
Quinoa	13.14 ± 0.01	6.56 ± 0.12	2.02 ± 0.01	8.28 ± 0.77	68.89 ± 0.05	90.61 ± 0.02	9.39 ± 0.02
Wheat	11.80 ± 0.03	3.61 ± 0.11	1.67 ± 0.01	12.56 ± 0.24	72.59 ± 0.05	89.67 ± 0.30	10.33 ± 0.30
Peanut Oilcake	46.01 ± 0.02	10.64 ± 0.14	4.34 ± 0.01	13.69 ± 1.11	30.93 ± 0.06	91.92 ± 0.05	8.08 ± 0.05
Broccoli	3.57 ± 0.33	0.45 ± 0.07	0.94 ± 0.07	4.72 ± 0.62	4.80 ± 0.08	9.76 ± 0.07	90.24 ± 0.07
Beets	2.39 ± 0.47	0.13 ± 0.01	1.27 ± 0.06	2.80 ± 0.45	10.99 ± 0.34	14.78 ± 0.44	85.22 ± 0.44

Values are mean ± SEM; Nitrogen to protein factors used was 6.25 for (quinoa, peanut oilcake, broccoli and beets) and 5.8 for wheat. Dry matter, DM., Digestible carbohydrate (% DM) = [100 − (Protein + Fat + dietary fiber + Ash)], *n* = 3. Dietary fiber from USDA nutrient base data.

**Table 2:** Dough Composition Flatbreads containing Quinoa, Wheat and Peanut Oilcake Flatbread Dough with Broccoli or Beets.

Flatbreads	Quinoa g	Wheat g	Peanut Oilcake, g	Broccoli g	Beets g	Salt, g	Water, mL/100 g Ingredients
QPCBROC	36.10	__	36.10	27.07	--	0.73	50.7
WPCBROC	--	36.10	36.10	27.07	--	0.73	54.1
QPCBEET	36.10	--	36.10	--	27.07	0.76	36.4
WPCBEET	--	36.10	36.10	--	27.07	0.73	36.10

Quinoa Peanut Oilcake Broccoli (QPCBROC), Wheat Peanut Oilcake Broccoli (WPCBROC), Quinoa Peanut Oilcake Beet (QPCBEET), Wheat Peanut Oilcake Beet (WPCBEET). Level of ingredients was decided by consensus of laboratory personnel determined as tasty flatbreads. Dough was set at room temperature for 30 min.

**Table 3:** Sensory Evaluation of Quinoa, Wheat and Peanut Oilcake with Broccoli or Beets Flatbreads.

Flatbreads	Color/ Appearance	Odor/ Aroma	Taste/ Flavor	Texture/ Mouth Feel	Acceptance
QPCBROC	4.25 ± 0.09 a	4.04 ± 0.09 a	3.80 ± 0.12 a	3.59 ± 0.11 a	1.83 ± 0.04 a
WPCBROC	3.78 ± 0.09 b	3.74 ± 0.10 bc	3.59 ± 0.12 ab	3.43 ± 0.11 ab	1.70 ± 0.05 a
QPCBEET	4.14 ± 0.09 a	3.95 ± 0.09 ab	3.40 ± 0.12 b	3.40 ± 0.12 ab	1.78 ± 0.05 a
WPCBEET	4.05 ± 0.10 a	3.54 ± 0.10 c	3.38 ± 0.12 b	3.16 ± 0.12 b	1.69 ± 0.05 a

Quinoa Peanut Oilcake Broccoli (QPCBROC), Wheat Peanut Oilcake Broccoli (WPCBROC), Quinoa Peanut Oilcake Beet (QPCBEET), Wheat Peanut Oilcake Beet (WPCBEET). Values (mean ± SEM) within columns with different letters differ significantly (*p* ≤ 0.05), *n* = 80. Sensory evaluation parameters were on a scale of 1–5 (Dislike very much = 1, dislike slightly = 2, neutral = 3, like slightly = 4, like very much = 5); Acceptance was on scale of 1–2 (Unacceptable = 1 and Acceptable = 2).

**Table 4:** Water Activity (Aw), True Density (ρt), Bulk Density (ρb), Porosity, Expansion, Break Force and Stretchability of Quinoa, Wheat and Peanut Oilcake with Broccoli or Beets Flatbreads.

Flatbreads	Aw, ERH/100	ρ*t*, kg/m^3^	ρb, kg/m^3^	Porosity, %	Expansion, %	Break Force, g	Stretchability, mm
QPCBROC	0.94 ± 0.01 a	1.40 ± 0.01 a	0.78 ± 0.01 b	0.44 ± 0.02 a	1.81 ± 0.05 a	1150 ± 110 c	7.31 ± 0.61 a
WPCBROC	0.94 ± 0.01 a	1.43 ± 0.01 a	0.90 ± 0.01 a	0.38 ± 0.01 b	1.6 0± 0.02 b	1341 ± 169 c	6.67 ± 0.80 a
QPCBEET	0.92 ± 0.01 b	1.44 ± 0.01 a	0.93 ± 0.03 a	0.35 ± 0.01 bc	1.55 ± 0.03 bc	2189 ± 91 a	4.25 ± 0.34 b
WPCBEET	0.94 ± 0.01 a	1.41 ± 0.01 a	0.94 ± 0.01 a	0.33 ± 0.01 c	1.50 ± 0.01 c	1802 ± 65 b	7.30 ± 0.20 a

Quinoa Peanut Oilcake Broccoli (QPCBROC), Wheat Peanut Oilcake Broccoli (WPCBROC), Quinoa Peanut Oilcake Beet (QPCBEET), Wheat Peanut Oilcake Beet (WPCBEET). Values (mean ± SEM) within columns with different letters differ significantly (*p* ≤ 0.05).

**Table 5:** Color parameters (L*, a*, b*) of flatbread samples.

Flatbreads	L* (Black = 0, White =100)	a* (Green −, Red +)	b* (Blue −, Yellow +)
QPCBROC	55.97 ± 0.59 b	7.29 ± 0.66 c	27.60 ± 0.42 a
WPCBROC	57.40 ± 0.33 a	5.34 ± 0.34 d	23.27 ± 0.45 b
QPCBEET	36.40 ± 0.18 d	24.85 ± 0.23 b	14.38 ± 0.44 c
WPCBEET	40.11 ± 0.42 c	26.76 ± 0.07 a	9.44 ± 0.35 d

Quinoa Peanut Oilcake Broccoli (QPCBROC), Wheat Peanut Oilcake Broccoli (WPCBROC), Quinoa Peanut Oilcake Beet (QPCBEET), Wheat Peanut Oilcake Beet (WPCBEET). Values (mean ± SEM) within columns with different letters differ significantly (*p* ≤ 0.05), *n* = 5.

**Table 6:** Chemical Composition of Flatbread Samples, DM basis.

Flatbreads	Protein	Fat	Minerals	Dietary Fiber	Carbohydrates	DM
QPCBROC	32.82 ± 0.01 a	5.80 ± 0.03 a	4.40 ± 0.01 a	11.41 ± 0.48 b	56.98 ± 0.02 c	64.70 ± 0.29
WPCBROC	30.58 ± 0.06 c	4.70 ± 0.03 b	4.25 ± 0.02 c	13.32 ± 0.38 a	60.47 ± 0.04 a	65.56 ± 0.46
QPCBEET	31.95 ± 0.05 b	5.34 ± 0.39 ab	4.35 ± 0.01 b	10.71 ± 0.45 c	58.36 ± 0.15 b	68.74 ± 0.27
WPCBEET	29.55 ± 0.02 d	5.15 ± 0.07 ab	4.13 ± 0.01 d	13.14 ± 0.35 a	61.17 ± 0.03 a	66.35 ± 0.08

Quinoa Peanut Oilcake Broccoli (QPCBROC), Wheat Peanut Oilcake Broccoli (WPCBROC), Quinoa Peanut Oilcake Beet (QPCBEET), Wheat Peanut Oilcake Beet (WPCBEET). Values (mean ± SEM) within columns with different letters differ significantly (*p* ≤ 0.05), *n* = 3. Dietary fiber calculated from Table 1 and Table 2 data. Values (mean ± SEM) within columns with different letters differ significantly (*p* ≤ 0.05), *n* = 3. Digestible carbohydrate (% DM) = [100 − (Protein + Fat + dietary fiber + Ash)].
